# Hepatocellular adenoma: comparison between real-time contrast-enhanced ultrasound and dynamic computed tomography

**DOI:** 10.1186/s40064-016-2406-z

**Published:** 2016-06-30

**Authors:** Wei Wang, Jin-Ya Liu, Zheng Yang, Yue-Feng Wang, Shun-Li Shen, Feng-Lian Yi, Yang Huang, Er-Jiao Xu, Xiao-Yan Xie, Ming-De Lu, Zhu Wang, Li-Da Chen

**Affiliations:** Department of Medical Ultrasonics, Institute of Diagnostic and Interventional Ultrasound, The First Affiliated Hospital of Sun Yat-Sen University, Guangzhou, China; Department of Pathology, The First Affiliated Hospital of Sun Yat-Sen University, Guangzhou, China; Department of Hepatobiliary Surgery, The First Affiliated Hospital of Sun Yat-Sen University, Guangzhou, China; Department of Radiology, The First Affiliated Hospital of Sun Yat-Sen University, Guangzhou, China; Department of Medical Ultrasonics, The Third Affiliated Hospital of Sun Yat-Sen University, Guangzhou, China

**Keywords:** Hepatocellular adenoma, Contrast-enhanced ultrasound, Dynamic computed tomography

## Abstract

**Purpose:**

To investigate and compare the contrast-enhanced ultrasound (CEUS) features of histologically proven HCA with those of contrast-enhanced computed tomography (CECT).

**Methods:**

Eighteen patients with proven hepatic adenoma by pathology were retrospectively selected from the CEUS database. Fourteen of them had undergone liver CECT exams. The basic features on unenhanced imaging and the enhancement level and specific features on contrast-enhanced imaging were retrospectively analyzed, and the differences between CEUS and CECT were compared.

**Results:**

All the HCAs showed hyper-enhancement in the arterial phase. During the portal and late phases, 12 HCAs (12/18, 66.7 %) on CEUS and 11 (11/14, 78.6 %) on CT showed washout. On CEUS, 10 (10/18, 55.5 %) showed centripetal filling in the arterial phase and persistent peripheral rim enhancement. Five of them (61.1 %, 11/18) showed delayed central washout in the portal or late phase. However, on CECT, 2 (14.3 %, 2/14) and 4 (28.6 %, 4/14) HCAs showed persistent enhancement of the peripheral rim and central non-enhancing hemorrhage areas, respectively.

**Conclusions:**

Compared with dynamic CT, CEUS was superior at characterizing specific dynamic features. Considering that it is radiation-free, readily availability and easy to use, CEUS is suggested as the first line imaging tool to diagnose HCA.

## Background

Hepatic adenoma (HCA) is the third most common benign hepatic neoplasm. It may undergo malignant transformation and has a marked tendency to hemorrhage (Laumonier et al. 2008). The differential diagnosis with other focal liver lesions (FLLs), particularly focal nodular hyperplasia (FNH) and well-differentiated hepatocellular carcinoma (HCC), is of great significance because of the different management and outcomes for patients (Bartolozzi et al. 1997; Dietrich et al. 2005; Kim et al. 2008; Roche et al. 2015; Kong et al. 2015; Lizardi-Cervera et al. 2006). Therefore, a non-invasive diagnosis is beneficial for further treatment.

Because of its broad availability and a faster multi-row detector, computed tomography (CT) has become an excellent tool for the detection and characterization of FLLs. Typical enhancements of HCA on CT are likely to be iso-attenuated with the surrounding liver on unenhanced CT, becoming hyper-attenuated in the arterial phase and then fading to iso-/hypo-attenuation in the portal or late phase (Hussain et al. 2006; Ichikawa et al. 2000). Hemorrhage and calcification in HCA are present at low sensitivities of 9–15.7 and 5–15 %, respectively (Hussain et al. 2006; Ichikawa et al. 2000). However, these imaging features are not specific to the diagnosis of HCA (Ronot and Vilgrain 2014). Furthermore, considering the radiation hazard, characterization of HCA using CT is a questionable practice.

Contrast-enhanced ultrasound (CEUS) has provided a real-time technique to delineate lesions (Bartolotta et al. 2009). It is believed that CEUS can depict more morphologic features of FLLs owing to its high spatial and temporal resolution (Wang et al. 2013; Claudon et al. 2013). On CEUS, in addition to centripetal enhancement in the arterial phase, persistent peripheral rim enhancement and delayed central washout are also reported as specific dynamic features for the characterization of HCA (Kong et al. 2015; Roche et al. 2015). Because CEUS is radiation-free, readily available and easy to use, many centers have employed CEUS as a routine part of their work-up of FLLs (Jung et al. 2007).

To date, many studies have focused on the differential diagnosis of HCA and FNH using CEUS (Dietrich et al. 2005; Kim et al. 2008; Kong et al. 2015). However, few studies have compared the characteristics of HCA on CEUS with those on dynamic CT. The purpose of our study was to compare the CEUS features of histologically proven HCA with those of CT.

## Methods

### Patient population

This retrospective study was approved by the ethics committee of the first and third hospital of Sun Yat-Sen University, and informed consent was obtained from all subjects. From June 2008 to October 2015, 18 patients (9 men and 9 women; mean age ± SD, 33 years ± 8; age range 18–52 years) were histologically proven to have hepatic adenoma and were retrospectively selected from our database. The pathological diagnosis was obtained by specimens from ultrasound-guided percutaneous biopsy (n = 2) or surgical resection (n = 16). Fourteen patients had undergone a dynamic CT of the liver. Their basic characteristics are presented in Table 1.

### CEUS techniques

All examinations were performed using the Aplio XV or 500 (Toshiba Medical Systems, Tokyo, Japan), equipped with a 375BT convex transducer (frequency range of 1.9–6.0 MHz), or the MyLab Twice (Esaote Medical Systems, Genoa, Italy), equipped with a CA541 convex transducer (frequency range of 1.0–8.0 MHz). The contrast-specific imaging modes used in the present study were contrast harmonic imaging (MI, 0.06–0.08) and contrast tuned imaging (MI, 0.06–0.10). After activation of the contrast mode, 2.4 ml of SonoVue (Bracco, Milan, Italy) were administered intravenously in a bolus fashion and flushed by 5 ml of 0.9 % saline solution. The target lesion was observed continuously for 6 min, and the entire arterial, portal and late phases were stored on the hard disk. The arterial, portal and late phases were defined as 0–30, 31–120 and 121–360 s after injection, respectively. All the CEUS examinations were performed by two experienced radiologists (W.W. and X.Y.X), both of whom had more than 8 years of experience in liver CEUS.

### CT techniques

Among 18 HCAs, 14 were analyzed with CT (Aquilion 64, Toshiba Medical System, Tokyo, Japan) within 2 weeks before or after the CEUS examination. The standard dynamic contrast-enhanced scan procedure is as follows: After an unenhanced helical sequence scan through the liver, 80–100 ml (1.5 ml/kg) of a contrast agent (Ultravist 300, Schering, Berlin, Germany) were administered via the antecubital vein at a rate of 3–4 ml/s. The following CT acquisition parameters were used: 120 kV; 200–250 mAs; collimation: 64 mm × 0.5 mm; slice thickness: 0.5 mm; slice increments: 0.5 mm; and pitch: 0.9. The arterial, portal and late phases were defined as 0–45, 46–120 and 121–360 s after injection, respectively.

### Image analysis

CEUS and dynamic CT images were retrospectively analyzed in consensus by two investigators (Z.W. and L.D.C.), both of whom had more than 6 years of experience evaluating liver imaging. They were not involved in the US or CT scanning and were unaware of the clinical and imaging information of the patients. The enhancement level of each phase and specific enhancement pattern were evaluated. The level of enhancement was subdivided into hypo-enhancement, iso-enhancement, and hyper-enhancement compared with the surrounding liver parenchyma. Centripetal filling was defined as rapid centripetal progression of the enhancement in the arterial phase. Persistent peripheral rim enhancement was defined as persistent hyper-echogenicity at the rim of the lesion in the arterial, portal and late phases. Central washout was defined as hypo-enhancement in the center of the lesion compared with the peripheral region (Claudon et al. 2013).

### Statistical analysis

The statistical analysis was performed using SPSS 16.0 software (SPSS Inc., Chicago, IL, USA). Data were presented as the mean ± standard deviation (SD). P < 0.05 was considered to indicate statistical significance. The detection rate of imaging features between CEUS and dynamic CT was assessed using χ^2^ or Fisher’s exact test.

## Results

### Basic features on unenhanced imaging

There were 6 (6/18, 33.3 %); 9 (9/18, 50.0 %); and 3 (3/18, 16.7 %) lesions located in the left, right and both lobes of the liver, respectively. The mean size of the lesions was 8.4 ± 5.5 cm (range 1.4–20 cm). All lesions were round shaped with clear boundary. On gray scale ultrasonography, 9 of 18 lesions (9/18, 50.0 %) appeared hypoechoic to the background liver parenchyma, and 6 (6/18, 33.3 %); 2 (2/18, 11.1 %); and 1 (1/18, 5.6 %) lesions appeared hyperechoic, isoechoic and mixechoic, respectively. On color Doppler, a short, rod-like flow pattern in HCAs or periphery vessels of the lesions was detected (8/18, 44.4 %). On the CT scan, 12 of 14 lesions (85.7 %) appeared hypo-attenuated to the background liver parenchyma, and only 1 lesion (1/8, 12.5 %) appeared hyper-, iso- and mix-attenuated, respectively (Table 1).

**Table 1:** Patient characteristics

Case No.	Age	Gender	Location	Diameter (cm)	US	CT	CEUS patterns	Dynamic CT patterns
1	37	F	S7	2.0	Iso	Hypo	Hype-Hype-Hype	Hype-Iso-Iso
2	30	F	S6.7	9.9	Hypo	Hypo	Hype-Hypo-Hypo	Hype-Hypo-Hypo
3	41	M	S6.7	5.4	Hypo	Hypo	Hype-Hypo-Hypo	Hyper-Iso-Iso
4	29	F	S5.6.7.8	17.2	Hype	Hypo	Hype-Hypo-Hypo	Hype-Hypo-Hypo
5	22	F	S4.5.8	20.0	Hypo	Hype	Hype-Iso-Hypo	Hype-Hype-Hype
6	35	F	S4	6.7	Hype	Hypo	Hype-Iso-Hypo	Hype-Hypo-Hypo
7	31	M	S4.8	5.5	Hypo	Hypo	Hype-Iso-Hypo	Hype-Iso-Hypo
8	26	F	S4	8.0	Hypo	Hypo	Hype-Iso-Iso	Hype-Hypo-Hypo
9	31	F	S2.3	1.4	Hypo		Hype-Iso-Iso	
10	45	F	S4	8.3	Hypo		Hype-Hypo-Hypo	
11	29	F	S6	18.0	Hypo	Hypo	Hype-Iso-Iso	Hype-Hypo-Hypo
12	38	M	S7	5.0	Hyper		Hype-Hypo-Hypo	
13	52	M	S7	4.5	Iso	Hypo	Hype-Hypo-Hypo	Hype-Hypo-Hypo
14	18	M	S4	8.8	Hyper	Hypo	Hype-Hype-Hype	Hype-Hypo-Hypo
15	36	M	S2.3.4	12.8	Mix	Mix	Hype-Hype-Hypo	Hype-Hypo-Hypo
16	38	M	S6	3.7	Hypo	iso	Hype-Iso-Hypo	Hype-iso-iso
17	28	F	S6.7	8.0	Hyper	Hypo	Hype-Iso-Iso	Hype-Hype-Hypo
18	44	M	S4.5	4.4	Hypo	Hypo	Hype-Hypo-Hypo	Hype-Iso-Hypo

*CEUS* contrast-enhanced ultrasound, *Hype* hyper-enhancement, *Iso* iso-enhancement, *Hypo* hypo-enhancement

### Enhancement level in vascular phases

**Fig. 1:**
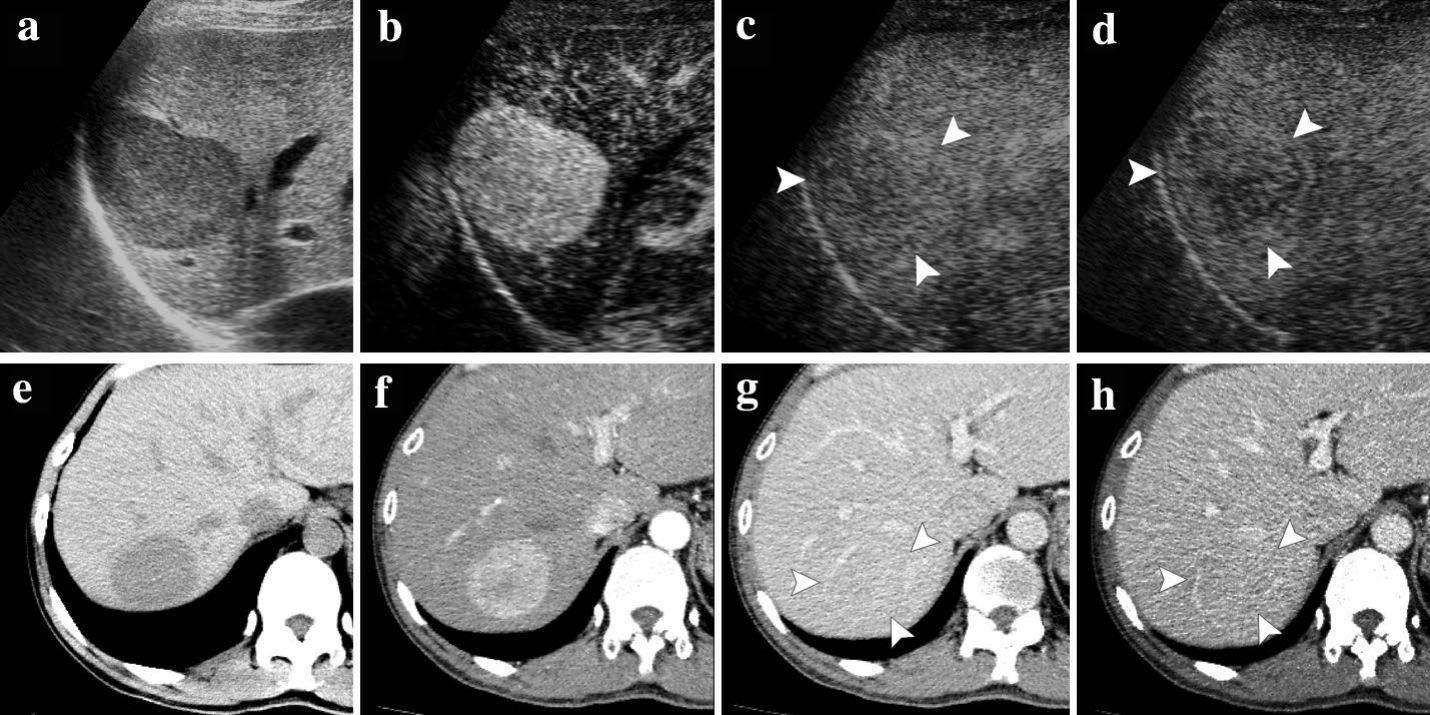
Contrast-enhanced ultrasound (CEUS) and dynamic computed tomography (CT) images of HCA in a 41-year-old female (Case 3) demonstrate a hypoechoic (**a**) and hypointense (**e**) lesion to the surrounding liver. On contrast-enhanced imaging, both modalities showed hyper-enhancement in the arterial phase (**b**, **f**). During the portal and late phases on CEUS, the lesion showed hypo-enhancement (**c**, **d**). However, the lesion is iso-enhanced (**g**, **h**)

On CEUS (n = 18) and dynamic CT (n = 14), all HCAs showed hyper-enhancement in the arterial phase. During the portal phase, 7 (7/18, 38.9 %) and 3 (8/14, 57.1 %) HCA showed hypo-enhancement on CEUS and dynamic CT, respectively. The washout continued on both imaging modalities in the late phase, with 12 HCAs (12/18, 66.7 %) on CEUS and 11 (11/14, 78.6 %) on CT showing hypo-enhancement. On both CEUS and dynamic CT, there were five enhancement patterns: “hyper-hypo-hypo”; “hyper-iso-hypo”; “hyper-iso-iso”; “hyper-hyper-hyper”; and “hyper-hyper-hypo” (Fig. 1, Table 2). No statistical significance was found among enhancement types on CEUS and dynamic CT (all *P* > 0.05).

**Table 2:** Imaging features on CEUS and dynamic CT

	CEUS (n = 18)	Dynamic CT (n = 14)	*P* value
*Enhancement types*			
Hype-Hype-Hype	11.1 % (2/18)	7.1 % (1/14)	0.596
Hype-Iso-Iso	22.2 % (4/18)	21.4 % (3/14)	0.649
Hype-Hype-Hypo	11.1 % (1/18)	7.1 % (1/14)	0.692
Hype-Iso-Hypo	22.2 % (4/18)	14.3 % (2/14)	0.460
Hype-Hypo-Hypo	38.9 % (7/18)	57.1 % (8/14)	0.252
*Enhancement patterns*			
Centripetal arterial filling	55.5 % (10/18)	0	0.001
Peripheral rim of persistent enhancement	61.1 % (11/18)	14.3 % (2/14)	0.009
Delayed central washout	27.8 % (5/18)	0	0.043
Central non-enhancing	16.7 % (3/18)	28.6 % (4/14)	0.351

*Hype* hyper-enhancement, *Iso* iso-enhancement, *Hypo* hypo-enhancement

### Specific features on contrast-enhanced imaging

**Fig. 2:**
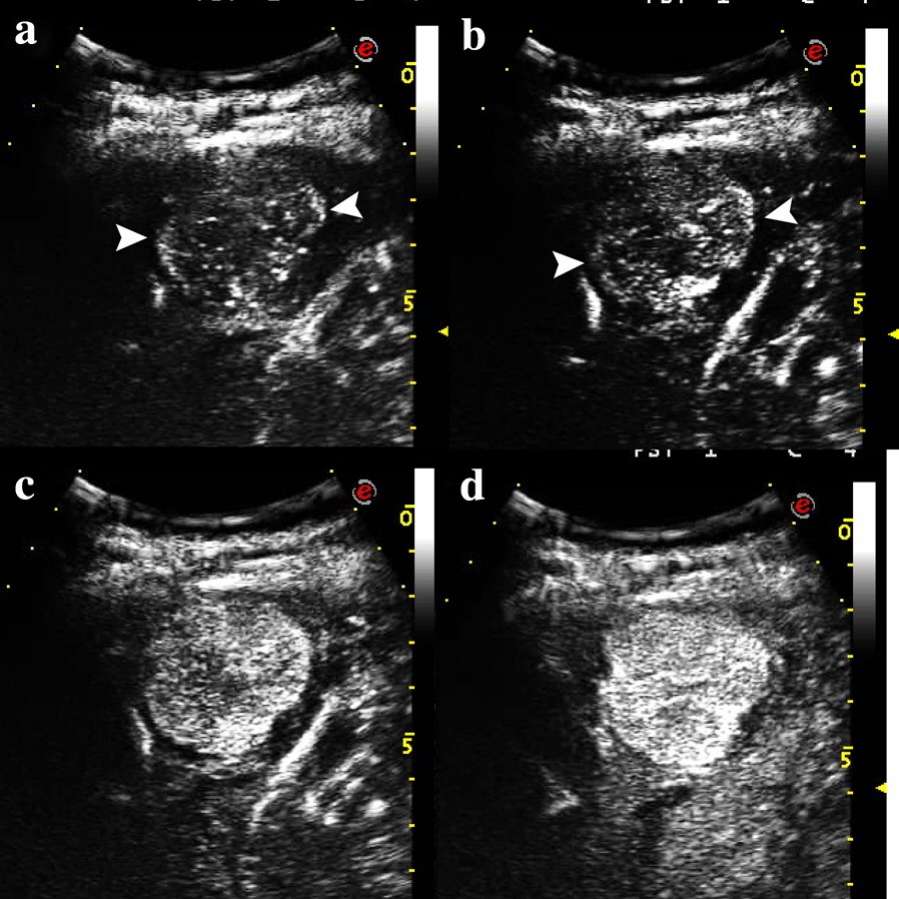
CEUS images in a 44-year-old male (Case 18) with HCA showed centripetal filling sign in the arterial phase. The lesion showed fine rim hyper-enhancement at about 11 s in the early arterial phase (**a**, **b**), following a rapid centripetal homogenous hyper-enhancement of whole mass (**c**, **d**)

**Fig. 3:**
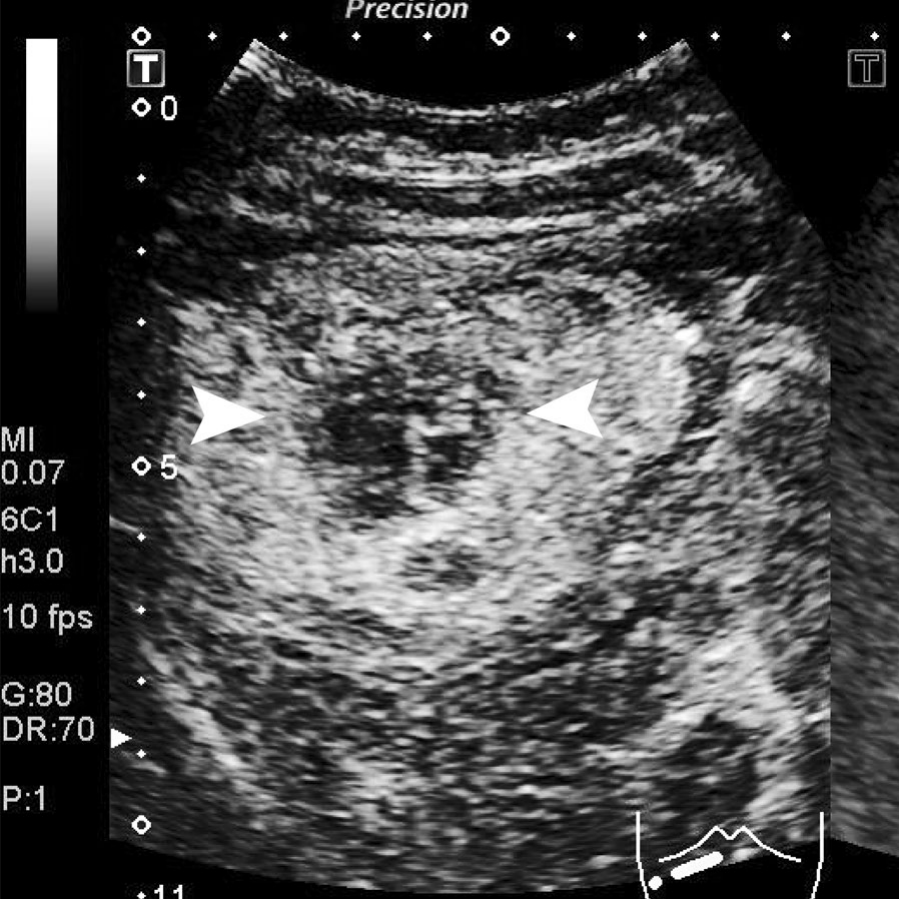
CEUS images in a 35-year-old female (Case 6) with HCA showed a central non-enhancing area indicating possible hemorrhage in the arterial phase

For the 18 HCAs with real-time CEUS, ten (10/18, 55.6 %) showed centripetal filling in the arterial phase (Fig. 2), and 11 (61.1 %, 11/18) showed persistent peripheral rim enhancement in the arterial, portal and late phases. Five (5/18, 27.8 %) showed delayed central washout in the portal or late phase. Three (3/18, 16.7 %) HCAs showed a central non-enhancing area that indicated a possible hemorrhage (Fig. 3). On CECT, 4 HCAs (4/14, 28.6 %) showed a central non-enhancing hemorrhage area. Specific features were more frequently detected on CEUS than on dynamic CT (all *P* < 0.05), except for central non-enhancement (*P* = 0.351) (Table 2). The vascular characteristics of the inflammatory HCAs and HNF-1 alpha mutated HCAs were summarized in Table 3.

**Table 3:** Imaging features of inflammatory and HNF1a-inactivated subtype of HCA on CEUS

	Inflammatory HCA (n = 6)	HNF1a-inactivated HCA (n = 3)
*Enhancement types*		
Hype-Hype-Hype	16.7 % (1/6)	0
Hype-Iso-Iso	33.3 % (2/6)	33.3 % (1/3)
Hype-Hype-Hypo	0	33.3 % (1/3)
Hype-Iso-Hypo	33.3 % (2/6)	0
Hype-Hypo-Hypo	16.7 % (1/6)	33.3 % (1/3)
*Enhancement patterns*		
Centripetal arterial filling	33.3 % (2/6)	33.3 % (1/3)
Peripheral rim of persistent enhancement	50.0 % (3/6)	33.3 % (1/3)
Central non-enhancing	33.3 % (2/6)	0

*Hype* hyper-enhancement, *Iso* iso-enhancement, *Hypo* hypo-enhancement

## Discussion

It has been reported that HCAs are most common in women who have taken oral contraceptives for long periods of time (Baum et al. 1973). The male-to-female ratio is 1:9, which is lower than that in our study (1:1). Our data coincide with reports from Asia (Kong et al. 2015; Hung et al. 2001) that suggested that a lower use of oral contraceptives in women and routine screening for hepatocellular carcinoma in men may result in a different male-to-female incidence ratio.

Several imaging modalities have been employed in the diagnosis of hepatic adenoma. However, no specific characteristics of HCAs can be identified on unenhanced imaging. Most HCAs are hypo-echoic or hyper-echoic on US and hypo-attenuated to the background liver parenchyma on CT. The mean size of HCAs in our study is larger than in previous reports (Zhu et al. 2011; Ricci et al. 2008). Although larger lesions are at a higher risk of hemorrhage, the echogenicity in our study were homogeneous. Unlike the spoke-wheel flow pattern of FNH, short rod-like or periphery vessels on color Doppler cannot proved effective diagnostic information for HCA (Kong et al. 2015).

On enhanced imaging, most reports showed that 45–60 % of HCAs were hyper-vascular in the arterial phase and hypo-enhanced in the portal or late phase. In our study, almost 70 % of lesions exhibited this enhancement pattern on CEUS and CECT, which can be encountered in HCC and hyper-enhancing metastases. Moreover, HCA with inhomogeneous enhancement is very difficult to discriminate from HCC in a non-cirrhosis liver. In case of this, future developments, such as elastography or new perfusion imaging, might be useful for classification of benign and malignant lesions (Wiggermann et al. 2013; Jung et al. 2007). On the other hand, 30.0 % of lesions on CEUS and 37.5 % on CECT appear to have sustained enhancement in later phases. Although this enhancement pattern suggests a benign tumor, a difficult differential diagnosis with FNH can also be encountered (Kong et al. 2015; Roche et al. 2015; Di Carlo et al. 2010; Kim et al. 2008; Dietrich et al. 2005; Bartolozzi et al. 1997).

This diagnostic dilemma could be resolved by specifically characterizing the dynamic imaging features. According to previous studies, HCAs have some special enhancement features, such as centripetal filling and persistent peripheral rim enhancement (Kim et al. 2008; Mathieu et al. 1986). The specific centripetal filling sign, which was discovered on arteriography, was first reported on CEUS by Kim (Kim et al. 2008). This is the main differential feature with FNH, which is characterized by centrifugal filling and central scarring. The detection rate ranges from 20 to 94 % (Laumonier et al. 2012; Kim et al. 2008; Kong et al. 2015), which is consistent with the rate of 55.5 % in our study. Laumonier reported that the inflammatory subtype of HCA had a peripheral rim of sustained enhancement with a hypo-echoic central area (Laumonier et al. 2012). In our study, this persistent peripheral rim enhancement feature was detected in 61.1 % of all HCAs and 50.0 % of inflammatory subtype on CEUS. The detection rate of this specific sign is lower in our cases because of the nine cases with unidentified subtype. However, none of the specific enhancement features discussed above was reported on CT. In our opinion, CEUS with inherent real-time scanning merit is better for display of those features. Although steatosis, calcification, necrosis and intro-tumor vessels on CT were reported in previous studies, these characteristics are not very sensitive (Burns and Wilson 2007; Hussain et al. 2006).

Our study has some limitations. First, it was a retrospective study based on small number of 18 cases, and only 14 of those were evaluated with CT. Second, as we focused on comparisons with CT, the problem of differential diagnosis with FNH or HCC was neglected. Therefore, limited information had been provided for the diagnostic accuracy of CEUS for this issue. Moreover, some imaging features vary depending on the subtype of HCA and/or tumor size. In our study, we failed to describe the correlation between imaging patterns and subtypes because of limited cases.

In conclusion, our study demonstrated that the enhancement level in three vascular phases of HCA on CEUS was consistent with that on CECT. Compared with CECT, the real-time capability of CEUS is superior for characterizing dynamic centripetal filling, peripheral rim of persistent enhancement, and delayed central washout. In addition, because it is radiation-free, readily availability, and easy to use, CEUS has been suggested as the first line imaging tool to diagnose HCA. Further studies using larger sample sizes and comparisons with MRI are required.
